# Effect of nutrition education intervention on nutrition knowledge, attitude, and diet quality among school-going adolescents: a quasi-experimental study

**DOI:** 10.1186/s40795-024-00850-0

**Published:** 2024-02-27

**Authors:** Sagar Raut, Dirghayu KC, Devendra Raj Singh, Raja Ram Dhungana, Pranil Man Singh Pradhan, Dev Ram Sunuwar

**Affiliations:** 1 Department of Nutrition and Dietetics, College of Applied Food and Dairy Technology, Purbanchal University, Lalitpur, Nepal; 2 Public Health Promotion and Development Organization, Kathmandu, Nepal; 3 School of Human and Health Sciences, University of Huddersfield, Huddersfield, UK; 4 Faculty of Medicine Nursing and Health Sciences, Monash University, Melbourne, Australia; 5 Department of Community Medicine, Maharajgunj Medical Campus, Institute of Medicine, Kathmandu, Nepal; 6 Department of Nutrition and Dietetics, Nepal Armed Police Force Hospital, Kathmandu, Nepal; 7 Department of Nutritional Science, University of Michigan School of Public Health, Ann Arbor, USA

**Keywords:** Adolescence, Attitude, Diet Quality, Knowledge, Nepal, Nutrition Education

## Abstract

**Background:**

Nutrition education is being used to encourage school adolescents to adopt healthy eating habits. To the best of our knowledge, very little study has been undertaken in Nepal to examine the effectiveness of nutrition education programs. This study aimed to assess the effect of nutrition education on nutritional knowledge, attitude, and diet quality among school-going adolescents in selected private schools in Nepal.

**Methods:**

A quasi-experimental study was conducted among 226 students aged 12 − 19 years of two selected private schools in Banepa municipality of Nepal. Students (*n* = 113) from the first school were assigned to intervention and the same number of students from the second school were enrolled in the study as the control. Over 12 weeks, students in the intervention group received one hour of nutrition education in the form of mini-lectures and interactive discussions, whilst students in the control group received no education. The student’s two-sample t-test was used to compare two groups and to assess the effectiveness of the nutrition education program.

**Results:**

Between the intervention and control group, the magnitude of difference in knowledge score was 1.80 (95% CI: 1.11 − 2.49), emotional eating was 0.98 (95% CI: 0.42 − 1.54), uncontrolled eating was 3.60 (95% CI: 2.10 − 5.09), and cognitive restraint of eating was 2.26 (95% CI: 1.51 − 3.01).

**Conclusions:**

A tailored health education intervention was found to be effective in increase nutritional knowledge and attitude among school-going adolescents. Adopting nutrition education interventions as part of public health school intervention builds positive knowledge, attitudes, and healthy eating habits in school-going adolescents.

**Supplementary Information:**

The online version contains supplementary material available at 10.1186/s40795-024-00850-0.

## Introduction

Adolescence is a critical time in a human’s life for promoting healthy choices and lifestyle behaviors to prevent risk factors of chronic diseases [1, 2]. Individuals aged between 10 and 19 years are considered adolescents [3]. The adolescent population constitutes 1.2 billion worldwide, making up 16% of the world’s population [4]. During adolescence, individuals are nutritionally vulnerable as their growth faces spurts of changes and if nutritional requirements are not met properly it might lead to malnutrition in later life [5]. To meet this increased demand, sufficient intake of both macro and micronutrients is crucial at this stage [6]. Nutritional knowledge plays an important role in influencing healthy food habits which ensures their nutrient needs during adolescence and later life [7, 8]. The basis for creating attitudes toward foods, nutrition, and health, as well as human eating behaviors, is nutrition knowledge [9]. To encourage adolescents to adopt healthy lifestyle practices, it is necessary to create a suitable environment at school and home [10]. Multiple components such as social, economic, and environmental factors must be considered to tackle childhood malnutrition [11].

However, evidence suggested that low education levels contributed to false perception, which was found to be one of the precipitating factors for nutritional deficiencies among adolescents [12]. People are more prone to changes in their lifestyle during adolescence, which may later affect their lives [13]. Understanding the benefits of good nutrition, and the value of a diverse diet as healthy eating habits at an early age could determine dietary practices later in the future [14]. As the schools have the potential of involving adolescent students, they are often considered for implementing education programs [15]. Educational program enhance knowledge, attitude, and practice for acquiring healthy behaviors among adolescents in schools or communities [16, 17].

Despite the efforts made by government and other agencies to promote healthy food choices in schools and public places, studies have shown that adolescents have poor knowledge, attitudes, and practices about malnutrition and dietary intake [8, 18]. It is often reported that adolescent in developing countries like Nepal has either lacked adequate nutrition knowledge or is influenced by misleading information to decide on healthy choices related to dietary habits [2, 19]. There is a significant increment in the adolescent adaptation to eating unhealthy food due to a lack of knowledge, unavailability, unaffordability, false perception of healthy foods, and socioeconomic boundaries [20, 21]. Today, quality diet choice among adolescent has drastically declined after the introduction of unhealthy fast food or processed food into the market [22].

The environment at school is the main target for conducting education programs as they involve a large number of children and adolescents [15]. Likewise, students may adopt and maintain healthy eating habits with the support of a nutrition education program that emphasizes the benefits of nutrition [21]. Nutrition education is a critical component of any strategy aimed at changing behavior and promoting healthy eating habits [23]. It is important to empower school students to cope with this tremendous burden and bridge the gap regarding the rising issue of falsification, as well as adolescent food choices and the health consequences that follow. This can only be achieved by providing them with proper nutrition knowledge, promoting healthy snack habits, and changing their overall attitudes toward healthy eating habits. The Government of Nepal runs the School Health Nutrition Program (SHNP) to improve the health and nutritional status of school-aged children [24]. However, very few comprehensive educational programs targeting the nutritional concerns of adolescents with the scientific assessment of nutrition knowledge, attitude, and dietary habits are being implemented in Nepal. Therefore, this study aimed to investigate the effect of a nutrition education intervention on nutritional knowledge, attitude, and diet quality among school-going adolescents in selected private schools located in the urban settings of Nepal.

## Methods

### Study design and setting

This school-based quasi-experimental study was conducted among 12 − 19 years school-going adolescents in grades 6 to 10 in two private schools in Banepa Municipality, Kavrepalanchok district of Nepal. Banepa is located about 25 km to the east of Kathmandu the capital city of Nepal. This study was conducted between February 2020 and July 2020. The study was reported using Transparent Reporting of Evaluations with Non-randomized Designs (TREND) guidelines [25].

### Participants

**Fig. 1:**
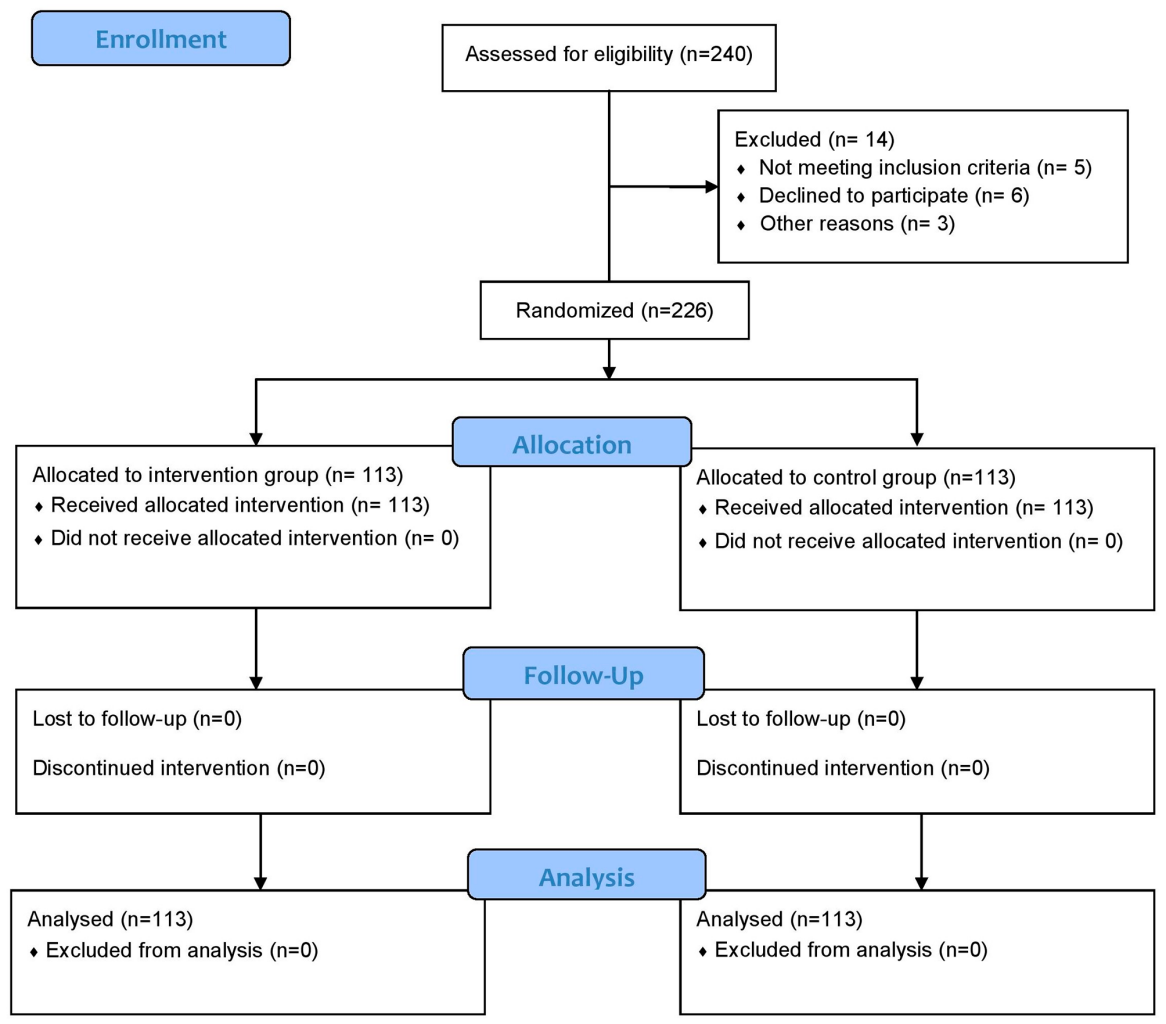
Flow diagram of the study

Study participants included 226 school-going adolescents, aged 12 − 19 years who were studying in grades 6 to 10 (Fig. 1). Those students who were able to answer the questionnaire items from grade 6 to grade 10 were present on the day of data collection were included in this study. Adolescents with medical conditions, such as physical and psychological issues, as well as those who were reluctant to participate and were absent on program days, were excluded from the study.

### Outcomes

The primary outcome of this study was to measure the change in nutrition knowledge of participants after a 12-week nutrition educational intervention, and the secondary outcomes were to evaluate change in nutrition attitude and diet quality among school-going adolescents.

### Intervention

In the intervention group, students were assigned to receive a nutrition education package, whereas the control group received no instruction regarding nutrition education, and they responded to questions based on regular health and nutrition education dissemination through the school curriculum/textbook. Follow-up data were collected after 12 weeks of nutrition education intervention. As per the nutrition education package, we provided 45 min of nutrition education sessions a day per week for intervention group in each grade (grade 6–10). Therefore, the students in the different grades received nutrition education session separately.

During the intervention phase, trained nutritionists/dietitians provided nutrition education to the intervention group using educational materials such as posters, leaflets, pamphlets, educational videos, and PowerPoint. The education package was developed by academic researchers and all materials reviewed by a multi-disciplinary expert group consisting of an academician and a dietitian before the intervention. A one-hour mini-lecture and interactive discussion session were conducted in the classroom. All the educational activities were carried out in the respective classroom for each section. The nutrition education sessions lasted approximately one hour in each class for five days and were held at the time of enrollment. Information and educational materials (posters, leaflets, pamphlets, educational videos, and PowerPoint) were handed out at the end of each session. The control group on the other hand did not receive the education package.

The nutrition education package contained important information on healthy eating habits, eating disorders in adolescents, food labels, knowledge of a balanced diet, and the consequences of poor eating habits. The origins and key roles of macro-and micronutrients, as well as the recommended dietary intake of various food items for teenagers and the principles of my plate, were also highlighted during the nutrition education session [26]. Follow-up visits were undertaken every four weeks to ensure the progression of healthy eating habits and a better understanding of nutrition.

### Sample size

The sample size was determined using effect size estimations of the difference in means between two independent groups using nutrition knowledge as the primary outcome variable, with a significance level (two-tailed) of 0.05 and a statistical power of 90%. Based on a similar study conducted by Fahlman et al. (2008) [27] where, the proportion of the nutritional knowledge score in the intervention and control groups was 0.49 and 0.39, respectively. The final sample size was 226 (113 participants in the intervention group and 113 participants in the control group, respectively) with a 10% non-response rate. The sample size was calculated using a test comparing two means in Stata/MP version 14.1 (StataCorp LP, College Station, Texas).

### Recruitment

**Fig. 2:**
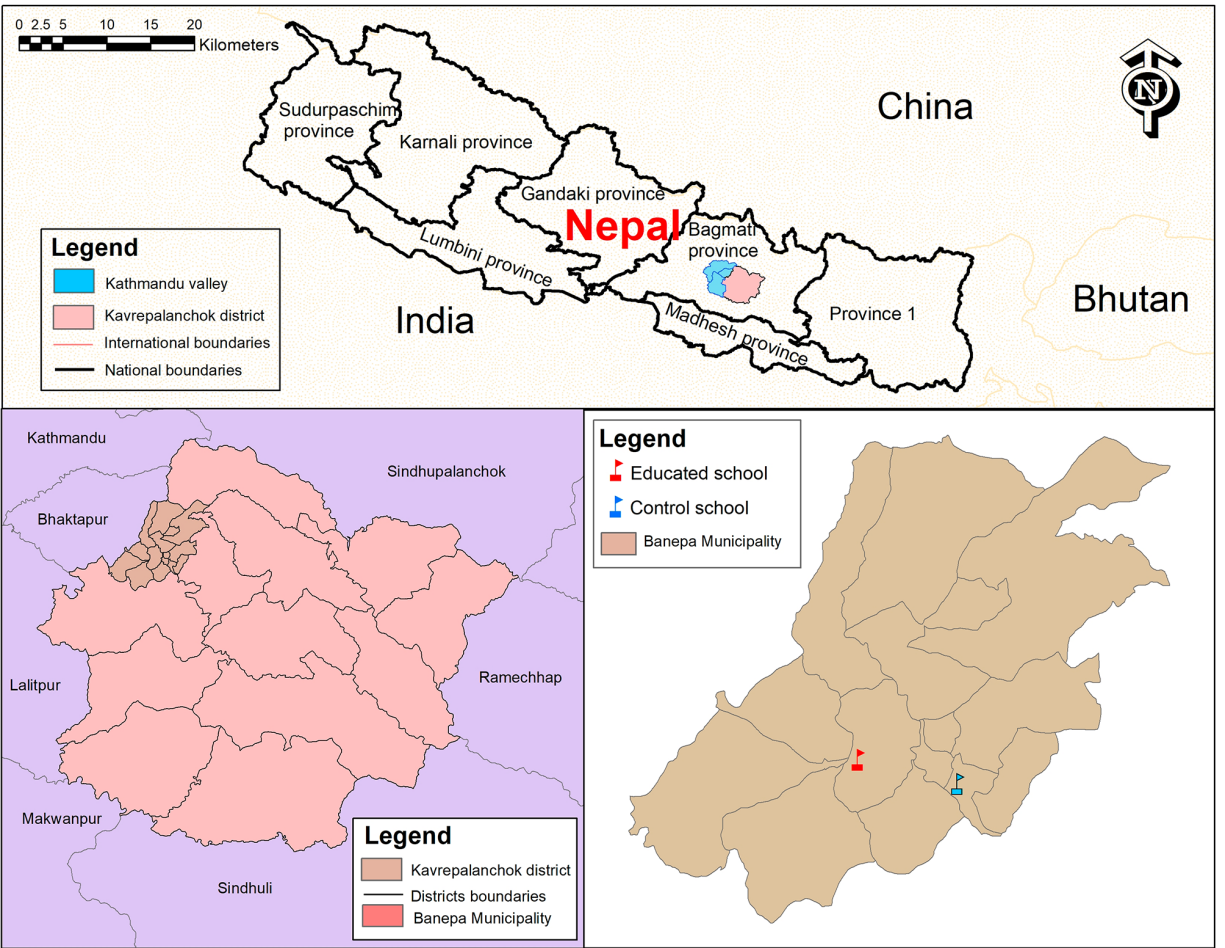
Map showing study area. The map was created using ArcGIS desktop version 10.8. The shape file of the administrative districts and location for Nepal was obtained from the Government of Nepal, Ministry of Land Management, and Survey Department website and was publicly available for unrestricted use

A list of private schools was obtained from Banepa Municipality. Each school has approximately 350 adolescents enrolled. Of 20 schools, only two private schools were chosen purposively chosen because the adolescent students in the two schools were adequate to meet the required sample size. The allocation of the intervention and control groups among the two schools was determined through the use of flipping a coin(Fig. 2). A complete list of sections/classes for each school was compiled with the help of a school’s administration registry. All students in grades 6 to 10 who were willing to participate in the study were enrolled individually from the two schools for both the intervention (*n* = 113) and control (*n* = 113) groups. The average number of students in each of the schools was around 350; however, the total number of students included in the study (*n* = 113 for each group) is less than the total number of students enrolled in the schools (*N* = 350). We approached all students to participate in the study, although the study sample size was determined to be 113 for each group. Among them, a few students dropped out due to loss of follow-up, unwillingness to give consent, absence on the day of enrollment, and illness. Thus, not all students were included in the study.

### Data collection tools and techniques

Data was collected by postgraduate (MSc. Nutrition and Dietetics) students who were provided with three days of training that included the objective of the study, data collection procedure, sampling method, ethical aspects of the study, and data entry techniques. Face-to-face interviews were conducted using pre-tested semi-structured questionnaires. Baseline interviews lasted almost 30 min in each school, and post-test data collection was conducted after 12 weeks of nutrition education intervention. All the tools were originally developed in the English language. Further, the tools were translated into the Nepali language and back translated into English to ensure the validity (and reliability) of the tool. Pretesting of the tools was carried out among 28 students from the neighboring school of Banepa Municipality.

The socio-demographic information includes age, sex, grade, religion, ethnicity, parent’s occupation, and parent’s education. The nutrition knowledge, attitude, and diet quality questionnaires were adapted from a previous study [21] and then translated into Nepali. The research committee and faculty members reviewed the pre-tested questionnaire to establish its validity and reliability. The questionnaire was revised appropriately based on their feedback. The Nepali version of the nutrition knowledge, attitude, and diet quality questionnaire had acceptable internal consistency (Cronbach’s alpha of 0.62, 0.72, and 0.61, respectively). However, the internal consistency of a score/scale is good at ≥ 0.7, and findings of ≥ 0.6 have also been deemed satisfactory or acceptable [28, 29].

Nutrition knowledge levels were determined based on eighteen questions (Q1 − Q18). The short form of the Food Frequency Questionnaire for Polish Children (SF-FFQ4PolishChildren) was adopted first in English and developed by Whati et al. [30], then translated into Nepali and modified to fit the Nepalese context. Correct answers were scored one point, while wrong or “don’t know” answers and missing data were given zero. The points of each respondent were totaled and examined further.

Attitudes toward nutrition were determined using a three-factor eating questionnaire (TFEQ13) designed specifically for youngsters. We utilized a simplified version of the questionnaire (TFEQ10) used by Hamulka et al. [21]., which consisted of only ten statements that further modified and verified the questionnaire for Nepalese settings. Three subscales, in a shortened version, were developed: emotional eating (Q1, Q3), Uncontrolled Eating (Q2, Q4, Q5, Q6, Q8), and Cognitive Restraint of Eating (Q7, Q9, Q10). Each subscale’s score was determined as the total of points allocated to respondents’ responses. All the responses were scored such that the responder may pick one of four options for each question: “definitely yes” (3 points), “rather yes” (2 points), “rather not” (1 point), and “absolutely not” (0 points).

Participants were asked to specify their usual frequency of consumption for the past 12 months for breakfast and school meals and nine food items such as dairy products, fish, vegetables, fruits, fruits or mixed fruit-veggie juices, fast foods, sweetened carbonated drinks, energy drinks, and sweets or confectionery [21]. It consisted of 11 questions. The diet quality scores were used for pro-Healthy Diet Index (pHDI), which were obtained by adding the daily frequencies of specific food items (as previously described) and expressing them as a percentage (range: 0 to 100). The diet quality scores were established (a priori approach) based on usual food frequency consumption within the last 12 months [21].

### Data management and analysis

The collected data were entered into EpiData software 3.1v and transferred into Stata/MP version 14.1 (StataCorp LP, College Station, Texas) for statistical analysis. The Chi-squared test was used to compare socio-demographic factors in the intervention and control groups at baseline. The student’s two-sample t-test was used to compare post-intervention magnitude of changes in nutrition knowledge, nutrition attitude, and diet quality scores between the intervention and control groups. To estimate changes between the control and intervention groups at baseline and follow-up, the Difference in Differences (DID) analysis was performed [31, 32]. We did not randomly assign the participants in the control and intervention group due to the nature of quasi-experimental study design. So, we used DID methods expecting to control heterogeneity between treatment and control group and achieve better exchangeability. In addition, there was a chance of both information and selection bias, which DID help minimize such bias by comparing changes in outcomes within each group. The statistical significance was considered at *p*-value < 0.05 and 95% confidence intervals (CIs).

### Ethical considerations

The ethical approval for this study was obtained from the Ethical Review Board (ERB) of the Nepal Health Research Council (Ref.no 1976). Formal permission was also obtained from the respective private schools. Written informed consent was obtained from all parents or legal guardians for eligible participants. The data collector also shared the objectives of the study with each participant before the baseline data collection and nutrition education. Participants and their parents or legal guardian were informed about voluntary participation, their right to refusal at any point, and the confidentiality of their identity.

## Results

A total of 226 participants were categorized into intervention (*n* = 113) and control (*n* = 113) groups. All the participants completed the study. The average (SD) age of the participants was 14.8 (1.2) years. There was no significant difference between the intervention and control groups in terms of socio-demographic variables such as age, sex, religion, and parental education. However, significant differences between these groups were observed in ethnicity, family type, and parental occupation (Table 1).

**Table 1:** Baseline characteristics of the study participants (*n* = 226)

Variables	Characteristics	Total sample (*n* = 226)	Intervention group (*n* = 113)	Control group(*n* = 113)	*p*-value^§^
		*n* (%)	*n* (%)	*n* (%)	
Age category	12–14	88 (38.9)	42 (47.7)	46 (52.3)	0.58
	15–17	138 (61.1)	71 (51.3)	67 (48.5)	
Ethnicity	Advantaged ethnic group	152 (67.3)	84 (55.3)	68 (44.7)	0.02*
	Disadvantaged ethnic group	74 (32.7)	29 (39.2)	45 (60.8)	
Sex	Male	136 (60.2)	66 (48.5)	70 (51.5)	0.58
	Female	90 (39.8)	47 (52.2)	43 (47.8)	
Religion	Hindu	202 (89.4)	105 (51.9)	97 (48.1)	0.08
	Non-Hindu	24 (10.6)	8 (33.3)	16 (66.7)	
Family type	Nuclear	94 (41.6)	37 (39.4)	57 (60.6)	< 0.01*
	Joint/extended	132 (58.4)	76 (57.6)	56 (42.4)	
Fathers’ education	Primary or lower	37 (16.4)	14 (37.8)	23 (62.2)	0.24
	Lower secondary	52 (23.0)	26 (50.0)	26 (50.0)	
	Higher secondary or above	137 (60.6)	73 (53.3)	64 (46.7)	
Mothers’ education	Primary or lower	52 (23.0)	23 (44.2)	29 (55.8)	0.59
	Lower secondary	73 (32.3)	39 (53.4)	34 (46.6)	
	Higher secondary or above	101 (44.7)	51 (50.5)	50 (49.5)	
Parents occupation	Job	52 (23.0)	34 (65.4)	18 (34.6)	0.04*
	Agriculture	54 (23.9)	24 (44.4)	30 (55.6)	
	Foreign employee/labor	120 (53.1)	55 (45.8)	65 (54.2)	

^§^denotes for Chi-squared test; *denotes statistically significant at p < 0.05

**Fig. 3:**
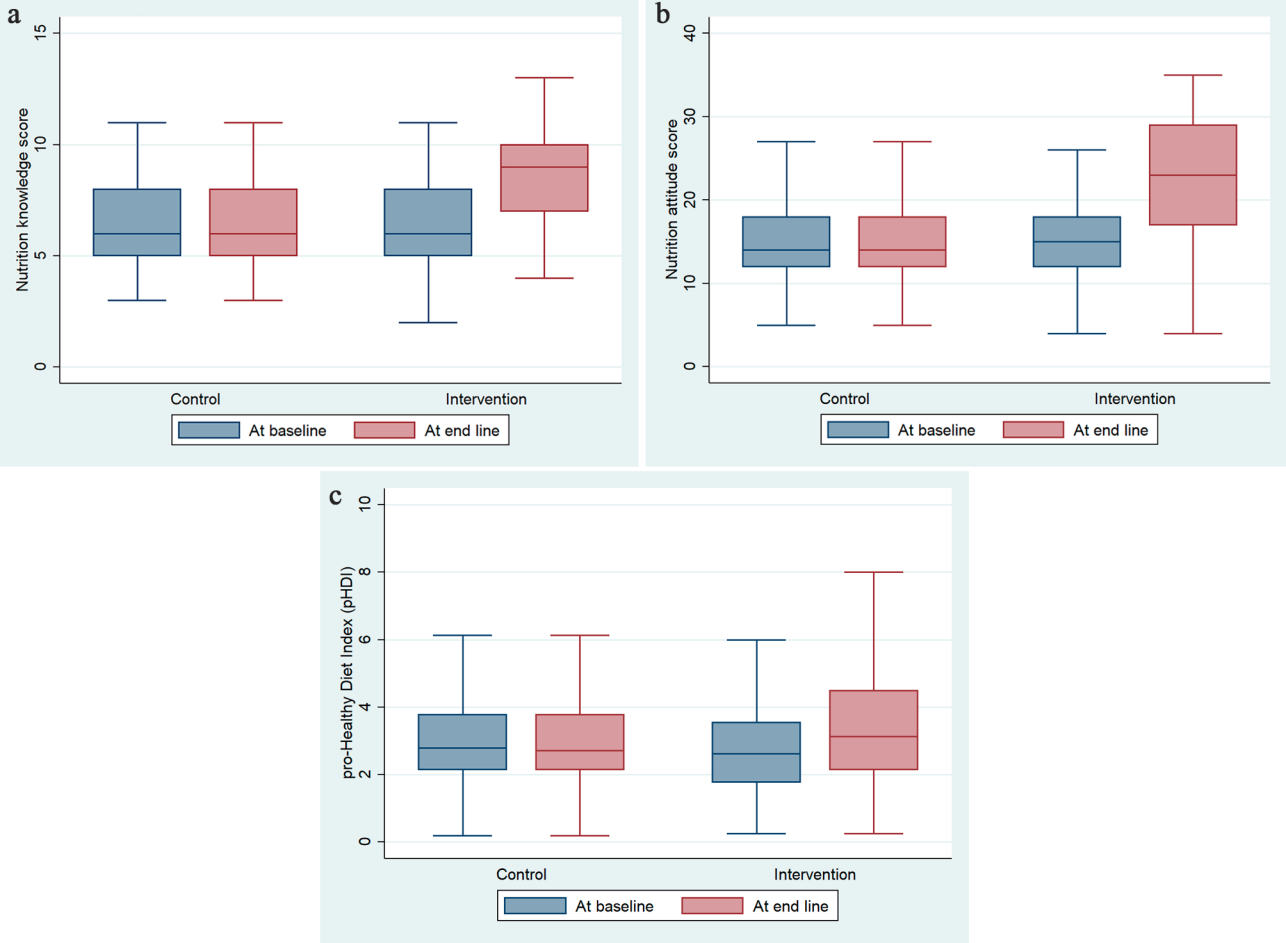
**a**. Effectiveness of education intervention on students’ nutrition knowledge. **b**. Effectiveness of education intervention on students’ nutrition attitude. **c**. Effectiveness of education intervention on students’ diet quality

In this study, Fig. 3a and b, and Fig. 3c depicted the comparison of nutrition knowledge, attitude, and diet quality scores, respectively between intervention and control groups at baseline and follow-up of the study. The intervention group had a higher median score than the control group at the follow-up. (Fig. 3a), (Fig. 3b) and (Fig. 3c).

At the end of 12 weeks of nutrition education intervention, the magnitude of changes in knowledge score between the intervention and control group was 1.80 with the nutrition education intervention (95% CI: 1.11 − 2.49). The difference was 0.98 (95% CI: 0.42 − 1.54) for emotional eating, 3.60 (95% CI: 2.10 − 5.09) for uncontrolled eating, and 2.26 (95% CI: 1.51 − 3.01) for cognitive restraint of eating (Table 2).

**Table 2:** Effectiveness of nutrition education on primary and secondary outcomes (*n* = 226)

Variables	Characteristics	Intervention group (*n* = 113)	Control group(*n* = 113)	*p*-value^†^	Difference in difference (DID)	*p*-value^‡^
		Mean (SD)	Mean (SD)		(95% CI)	
Nutrition knowledge score	Baseline	6.59 (2.04)	6.50 (1.76)	0.72	1.80 (1.11 − 2.49)	< 0.001
	Follow-up	8.69 (2.05)	6.81 (1.60)	< 0.001		
	Change	2.10 (0.55)	0.31 (0.54)	< 0.001		
**Nutrition attitude score**						
Emotional eating	Baseline	2.70 (1.43)	2.17 (1.41)	< 0.001	0.98 (0.42–1.54)	< 0.001
	Follow-up	3.75 (1.74)	2.47 (1.44)	< 0.001		
	Change	1.05 (0.65)	0.30 (0.46)	< 0.001		
Uncontrolled eating	Baseline	9.86 (3.87)	9.46 (3.80)	0.43	3.60 (2.10–5.09)	< 0.001
	Follow-up	13.46 (4.64)	9.81 (3.68)	< 0.001		
	Change	3.59 (1.43)	0.34 (0.49)	< 0.001		
Cognitive restrain	Baseline	2.91 (1.82)	3.34 (1.84)	0.07	2.26 (1.51–3.01)	< 0.001
	Follow-up	5.17 (2.51)	3.67 (1.85)	< 0.001		
	Change	2.26 (1.18)	0.32 (0.47)	< 0.001		
pro-Healthy Diet Index (pHDI)	Baseline	2.94 (1.75)	2.99 (1.47)	0.80	0.44 (-0.14-1.04)	0.14
	Follow-up	3.37 (1.70)	3.03 (1.54)	0.11		
	Change	0.43 (0.37)	0.03 (0.22)	< 0.001		

†*p*-value significance level difference for independent t-test; ^‡^denotes for *p*-value of intervention effect size which was measured using DID estimates from a linear regression model

## Discussion

This study demonstrated that educational intervention was effective to improve nutrition knowledge, attitude, and diet quality among school-going adolescents. As the burden of diet-related diseases continue to surge worldwide [33, 34], nutrition education interventions as part of public health school intervention may have implications to improve the overall nutritional status among school-going adolescents.

In the current study, there was a significant improvement in the mean nutrition knowledge score of 1.80 after the implementation of the intervention compared to the control group. This finding is in line with previous studies [16, 18, 35, 36]. Likewise, a systematic review based on Sub-Saharan Africa (SSA) provides evidence in favor of the positive impact of nutrition education on nutrition knowledge, where nutrition knowledge improved from 45.4 to 58.8% in the intervention group compared to the control group [37]. Furthermore, another study found that nutrition knowledge increased significantly soon after the nutrition education intervention and six months thereafter [38]. In addition, students with nutrition education intervention showed higher nutritional knowledge scores. The improvement in knowledge scores could be explained by the participants’ improved understanding and awareness regarding the role of a healthy diet and healthy eating behavior on their health and nutrition status [18]. As the burden of diet-related disorders continues to rise worldwide, investing in high-quality research to determine nutrition knowledge is a foresighted approach [33]. Nutrition education is used to encourage school adolescents to adopt healthy eating habits for the rest of their lives [18]. Also, successful nutrition education intervention should include content and teaching strategies that are suitable for the children and address change in the environment [39].

In our study, students in the intervention group had significantly improved emotional eating as well as cognitive restraint of eating scores. An increase in cognitive level leads to healthier eating and remarkably higher food and nutrition knowledge [40]. Evidence suggested that school-based health intervention programs could significantly influence eating behaviors and help adolescents adopt healthy eating practices [21, 41], which could be the reason for the significant improvement in the nutrition attitude of the participants of our study which might be connected with the way of presenting nutrition topics and the improvement in the learners’ nutrition knowledge, attitude as well as practice [33].

With the mixed results in the available literature [42, 43], the results of this study support changed lifestyle habits and improved diet quality of the targeted participants. Further to this another intervention study conducted observed no significant improvements in diet quality [44]. These inconsistent results might be contributed to the variety of approaches adopted, duration of intervention, efficacy, intervention quality, research ethics endorsed, and the overall methodology applied to achieve the desired objectives [42, 43]. Moreover, the poor availability of diverse food items due to seasonal factors might influence the practice of improved diet quality [45].

The strength of this study was the educational approach, which was relatively simple, short-term, and contextualized in a Nepalese setting thus making it feasible to implement. This study was using a quasi-experimental design that included both an intervention and a control group. This study included an analysis of the difference in difference using a linear regression model between the two groups. This information can be used to develop targeted nutrition education programs that are integrated into the academic curriculum. Despite its strength, this study had a few limitations. Since the study used quasi-experimental study design, participants were not randomly assigned that leads to lack of exchangeability. As the study participants were confined to two private schools in the urban area and the subsequent selection of the participants were made based on their willingness to participate in the study, study findings should cautiously be generalized to other settings. Likewise, information collected through face-to-face interviews might be prone to social desirability bias. However, we have included cross-validation questions e necessary in the tools to minimize the potential social desirability bias. In addition, compliance with nutrition education was not assessed in this study.

## Conclusions

This study concludes that the nutrition education intervention was effective to enhance the nutritional knowledge and attitude among school-going adolescents in Nepal. The findings of this study indicate that nutrition education in secondary schools is a good approach to improving adolescent nutrition knowledge, attitudes, and diet quality. Adopting a tailored nutrition education intervention program as part of government administered public health school intervention incorporating interactive discussions and knowledge of food habits is beneficial to building positive attitudes towards the importance of nutrition, enhancing nutrition knowledge, and supporting healthy eating in school-going adolescents.

### Electronic supplementary material

Below is the link to the electronic supplementary material.

Supplementary Material 1

Supplementary Material 2

Supplementary Material 3

## Data Availability

The datasets used during the current study are available from the corresponding author upon reasonable request.

## Abbreviations

CI: Confidence interval
CIs: Confidence intervals
DID: Difference in difference
DID: Difference in Differences
ERB: Ethical Review Board
pHDI: Pro-Healthy Diet Index
SF-FFQ4PolishChildren: Food Frequency Questionnaire for Polish Children
SHNP: School Health Nutrition Program
SSA: Sub-Saharan Africa
TFEQ: Three-factor eating questionnaire
TREND: Reporting of Evaluations with Non-randomized Designs
