# Supplementary material for: Effect of nutrition education intervention on nutrition knowledge, attitude, and diet quality among school-going adolescents: a quasi-experimental study
Source: BMC Nutr. 2024 Feb 27;10:35. doi: 10.1186/s40795-024-00850-0 (PMC10900745; doi:10.1186/s40795-024-00850-0)
Supplement: Supplementary file 1 — Supplementary Material 1 [file 40795_2024_850_MOESM1_ESM.docx]

## Supplementary file S1: Questionnaire

Study Questionnaire in English-Baseline/End line

Title of Thesis: **Effect of nutrition education intervention on nutrition knowledge, attitude, and diet quality among school-going adolescents: A quasi-experimental study**

Participant ID number………… Date (dd/mm/yy)……………

| Q.N | Questions | Response | code | Remark |
| --- | --- | --- | --- | --- |
| Section 1: Socio-Demographic characteristics. | | | | |
| 1 | Age | ……year |  |  |
| 2 | Sex | Male  Female | 1  2 |  |
| 3. | Cast/ethnicity | Brahmin  Chhetri  Dalit  Newar  Janajati  Other Specify.. | 1  2  3  4  5  6 |  |
| 4. | Religion | Hindu  Buddhist  Christian  Other specify… | 1  2  3  4 |  |
| 3. | Total members in family | No……. |  |  |
| 4. | Father education | No education  Primary  Some secondary  SLC and above | 1  2  3  4 |  |
| 5. | Mother education | No education  Primary  Some secondary  SLC and above | 1  2  3  4 |  |
| 6. | Parents occupation | GO  NGO/Private  Agriculture  Business  Foreign employee  Daily labor  Other(specify) | 1  2  3  4  5  6  7 |  |
| 7. | Monthly income of family | UptoRs. 2000  Rs. 2001-5000  Rs. 5001-10000  More than Rs. 10000 | 1  2  3  4 |  |

**Section 2: Nutritional Knowledge**

*To what extend do you agree with following statements? Please select one answer*

| **1. The key of healthy eating is:**  (1) to eat many different kinds of foods  (2) to eat more of some products than others  (3) to eat moderate or small amounts of some products  (4) all above answers  (5) I don't know | **2. How much of milk and milk beverages e.g. Dahi, Mohi and yoghurt should you eat during a day?**  (1) none  (2) half a glass  (3) one glass  (4) two glasses  (5) I don't know |
| --- | --- |
| **3. How much fruits and vegetables should you eat?**  (1) one fruit and one vegetable a day  (2) 3-4 fruits and one vegetables a day  (3)5or more fruits and one vegetables a day  (4) it is not necessary to eat fruits and vegetables  (5) I don't know | **4.”Good” microorganisms are such microorganisms which:**  (1) cause food fermentation  (2) cause bread rising  (3) have positive influence on human health  (4) all above answers  (5) I don't know |
| **5. Fast foods contain much of:**  (1) fat and fiber  (2) fat and salt  (3) fat and minerals  (4) protein and vitamins  (5) I don't know | **6. Which set of products contains much fiber?**  (1) whole-meal bread, apple, bean  (2) milk, yoghurt, cheese  (3) beef, poultry, pork  (4) butter, margarine  (5) I don't know |
| **7. Main sources of calcium in diet are:**  (1) dairy products  (2) vegetables and fruits  (3) dairy products, meat and meat products (4) cereal products  (5) I don't know | **8. Which breakfast set contains less fat?**  (1) sandwich with butter and half fat cottage cheese  (2) sandwich with margarine and cheese (3) corn flakes with full fat milk  (4) shortbread chocolate cookies with low fat yoghurt  (5) I don't know |
| **9. Main function of protein in the body is:** (1) structure  (2) energy  (3) regulation and structure  (4) energy and regulation  (5) I don't know | **10. Alkalizing products are:**  (1) cereal products  (2) fruits, vegetables and potatoes  (3) meat and meat products  (4) sugar and sweets  (5) I don't know |
| **11.** **Which information on the food label is most important for every consumer’s food safety?**  (1) ingredients  (2) nutrition value  (3) shelf life  (4) preservatives presence  (5) I don't know | **12.Vegan diet means that one is eliminating from diet:**  (1) meat, dairy and eggs  (2) meat and dairy  (3) meat  (4) vegetables and fruits  (5) I don't know |
| **13.Amount of human energy requirement depends on:**  (1) age and sex  (2) age, sex and physical activity (3) only physical activity  (4) only age  (5) I don't know | **14.BMI index is a ratio of:**  (1) body weight and height  (2) height and age  (3) body weight and age  (4) waist and height  (5) I don't know |
| **15.To be active means:**  (1) go to the gym  (2) walk often  (3) play sports e.g. football, volleyball, bike riding  (4) all above answers  (5) I don't know | **16. Which way of cooking is the best for vitamin C retention in potatoes?**  (1) cooking in a pot, starting from cold water  (2) cooking in a pot, starting from the boiling water  (3) pressure cooking in water  (4) steam cooking  (5) I don't know |
| **17.Where one should keep an open container of juice:**  **(1) in a cupboard**  **(2) on a table**  **(3) in special cupboard for fruits**  **(4) in refrigerator**  **(5) I don't know** | **18.Energy drinks are safe for adolescences:** (1) in limited amounts  (2) in unlimited amounts  (3) should not be consumed before age of 7 (4) should not be consumed before age of 16  (5) I don't know |

**Section 3: Nutrition Attitude questionnaire**

*Please fill out or circle one answer*

| **1.When I feel anxious, I find myself eating.**  (1) definitely yes  (2) rather yes  (3) rather no  (4) definitely no | **2. Being with someone who is eating often makes me hungry enough to eat also.**  (1) definitely yes  (2) rather yes  (3) rather no  (4) definitely no |
| --- | --- |
| 3. **When I feel blue, I often overeat**.  (1) definitely yes  (2) rather yes  (3) rather no  (4) definitely no | **4. When I see a real delicacy, I often get so hungry that I have to eat right away**. (1) definitely yes  (2) rather yes  (3) rather no  (4) definitely no |
| **5. I get so hungry that my stomach often seems like a bottomless pit**.  (1) definitely yes  (2) rather yes  (3) rather no  (4) definitely no | **6. I am always hungry so it is hard for me to stop eating before I finish the food on my plate.**  (1) definitely yes  (2) rather yes  (3) rather no  (4) definitely no |
| **7. I consciously hold back at meals in order not to gain weight.**  (1) definitely yes  (2) rather yes  (3) rather no  (4) definitely no | **8. I do not eat some foods because they make me fat**.  (1) definitely yes  (2) rather yes  (3) rather no  (4) definitely no |
| **9. I am always hungry enough to eat at any time.**  (1) definitely yes  (2) rather yes  (3) rather no  (4) definitely no | **10. How much you restraint the food intake? (circle the right number below)**     \| 1 \| 2 \| 3 \| 4 \| 5 \| 6 \| 7 \| 8 \| \| --- \| --- \| --- \| --- \| --- \| --- \| --- \| --- \|     On a scale of 1 to 8,  Where 1 means no restraint in eating and 8 means total restraint, what number would you give yourself?  1 – eat whatever I want, whenever I want it,  8 – constantly limiting food intake, never `giving in' |

**Section 4: Diet quality**

*Circle the best matching answer*

| **1. How often do you eat the first breakfast?**  (1) less than once a week  (2) 1-3 times a week  (3) 4-6 times a week  (4) every day | **2. How often do you eat a meal at school, e.g. second breakfast or lunch?**  (1) less than once a week  (2) 1-2 times a week  (3) 3-4 times a week  (4) every school day |
| --- | --- |
| **3. How often do you eat diary, e.g. milk, yoghurt, cottage cheese, cheese?**  (1) never or almost never  (2) less than once a week  (3) once a week  (4) 2-4 times a week  (5) 5-6 times a week  (6) every day  (7) few times a day | **4. How often do you eat fish e.g. baked, fried, smoked, marinated, canned?**  (1) never or almost never  (2) less than once a week  (3) once a week  (4) 2-4 times a week  (5) 5-6 times a week  (6) every day  (7) few times a day |
| **5. How often do you eat fast food, e.g. fries, pizza, hamburger, toasted sandwich?**  (1) never or almost never  (2) less than once a week  (3) once a week  (4) 2-4 times a week  (5) 5-6 times a week  (6) every day  (7) few times a day | **6. How often do you drink sweetened soft drinks (both carbonated and not) like cola, sprite, water with fruit syrup, ice tea?**  (1) never or almost never  (2) less than once a week  (3) once a week  (4) 2-4 times a week  (5) 5-6 times a week  (6) every day  (7) few times a day |
| **7. How often do you drink fruit or mixed fruit veggie juice e.g. apple, carrot-banana?**  (1) never or almost never  (2) less than once a week  (3) once a week  (4) 2-4 times a week  (5) 5-6 times a week  (6) every day  (7) few times a day | **8. How often do you drink energy drinks, e.g. Red Bull, Tiger, Burn or similar?**  (1) never or almost never  (2) less than once a week  (3) once a week  (4) 2-4 times a week  (5) 5-6 times a week  (6) every day  (7) few times a day |
| **9. How often do you eat vegetables, e.g. fresh, cooked, baked, and stewed?**  (1) never or almost never  (2) less than once a week  (3) once a week  (4) 2-4 times a week  (5) 5-6 times a week  (6) every day  (7) few times a day | **10. How often do you eat fruit e.g. fresh, frozen, as a part of a dish?**  (1) never or almost never  (2) less than once a week  (3) once a week  (4) 2-4 times a week  (5) 5-6 times a week  (6) every day  (7) few times a day |
| **11. How often do you eat sweets, e.g. cake, cookies, candies, bars, chocolate?**  (1) never or almost never  (2) less than once a week  (3) once a week  (4) 2-4 times a week  (5) 5-6 times a week  (6) every day  (7) few times a day | |
| **12. In the past week, how often did you eat breakfast?**  (1) Everyday  (2) 3-5 days per week  (3) 2 days or less per week  (4) Never | **13. In the past week, What type of lunch did you eat most day?**  (1) Packed Lunch from home  (2) I bought lunch outside of school  (3) School Lunch  (4) I didn’t have lunch at all |
| **14. In the past week, What type of lunch did you eat most day?**  (1) Homemade meal  (2) Take-out or fast food  (3) Meal at a restaurant  (4) I didn’t have dinner | **15. Do you snack between meals?**  (1) Yes  (2) No |
| **16. What type of snack do you eat between meals?**  (1) cakes, sweets, pastries  (2) chips, popcorn, pretzels, crackers  (3) Fruit or Veggies  (4) yogurt, cheese, milk  (5) Cereal bars  (6) Smaller meals | |

**Section 5: Knowledge on junk food consumption questionnaire**

| Questions | Response | Correct Response |
| --- | --- | --- |
| **Meaning of Junk Food** | | |
| Low nutrient value |  |  |
| High content of fat and sugar |  |  |
| Noodles and Pizza as most common type of junk food |  |  |
| Junk food offers high calorie in low volume |  |  |
| **Harmful Ingredient in Junk food** |  |  |
| Caffeine and phosphoric acid in cold drinks |  |  |
| Preservative in junk food |  |  |
| Use of MSG in junk food |  |  |
| **Effect of Harmful ingredients in junk food** |  |  |
| Effect of MSG in junk food |  |  |
| Effect of preservative in junk food |  |  |
| Effect of Caffeine and Phosphoric Acid in junk food |  |  |
| Effect of fat in junk food |  |  |
