# Supplementary material for: Effect of nutrition education intervention on nutrition knowledge, attitude, and diet quality among school-going adolescents: a quasi-experimental study
Source: BMC Nutr. 2024 Feb 27;10:35. doi: 10.1186/s40795-024-00850-0 (PMC10900745; doi:10.1186/s40795-024-00850-0)
Supplement: Supplementary file 2 — Supplementary Material 2 [file 40795_2024_850_MOESM2_ESM.docx]

**Supplementary file**

**Effect of nutrition education intervention on nutrition knowledge, attitude, and diet quality among school-going adolescents: A quasi-experimental study**

The aim of this study was to assess the effect of nutrition education on nutritional knowledge, attitude, and diet quality among school-going adolescents in selected private schools in Nepal. Participants were enrolled into two groups: intervention and control group. In the intervention group, students were assigned to receive a nutrition education package, whereas the control group received no education regarding nutrition knowledge, attitude, and practice. Students of control group responded to questions on the basis of regular health and nutrition education disseminated through school curriculum and text book, whereas in the intervention group, trained nutritionists/dietitians provided nutrition education using educational materials such as posters, leaflets, pamphlets, and PowerPoint. The education package was developed by principal investigator and all materials were reviewed by a multi-disciplinary expert group consisting of an academician and a dietitian before the intervention. A one-hour interactive discussion session was conducted in the classroom. All the education activities were carried out in the respective classroom for each section. The nutrition education sessions lasted approximately one hour in each class and were held at the time of enrollment. Information and education materials (posters, leaflets, pamphlets, educational videos and PowerPoints) were handed out at the end of each session. The control group on the other hand did not receive the education package.

The nutrition education package included mainly five important information: 1) healthy eating habits, 2) eating disorders in adolescents, 3) food labels, 4) knowledge of a balanced diet, and 5) the consequences of poor eating habits. Follow-up visits were undertaken once in an every four weeks, total of three times in twelve weeks to ensure the progression of healthy eating habits and a better understanding of nutrition.

Nutrition education package consists of two intervention phase. Firstly, group counseling which include one hour mini-lecture on each class (6-10) every week. Mini-lecture was prepared on PowerPoint under supervision of nutrition and dietitian. Group counseling consisted of five mini-lecture sessions, each session includes one topic and each session last for an hour a day per week in each class (6-10). Secondly, distribution of information and education materials (posters, pamphlets and leaflets) at the end of each session.

**Group counseling topics:**

1. **Healthy eating habits**: First session enhanced healthy dietary habits of school going adolescents. Topics under healthy eating habits were consumption of fruits and vegetables, managing sugar intake and avoiding processed foods, reducing salt and sodium intake, balance and proportion control were discussed through PowerPoint presentation followed by question and answer session.
2. **Eating disorders in adolescents**: The aim of this session was to identify signs of eating disorders and its management process. Three types of eating disorders (anorexia nervosa, bulimia nervosa, binge-eating disorder) were presented and discussed through presentation.
3. **Food labels**: This topic aims to impart useful information of food of our choice. In this session important things to look at on a food label (product name, date mark, net weight, and list of ingredients, nutrition information, name and address of manufacturer) were discussed and demonstrated by showing different food packing materials in order to better understanding of topic.
4. **Balance diet**: This session provides the knowledge regarding proper and balance nutrients to the body for normal functioning and growth. Components such as; carbohydrate, fats, proteins, vitamins, water and minerals were discussed through PowerPoint presentation.
5. **Consequences of poor eating habits**: In this session, various types of health effects of poor and unhealthy eating habits like; diabetes, heart disease, hypertension, stress/depression, obesity and lack of energy were talk and discussed by showing, poster and videos.

**Annex 1**

**
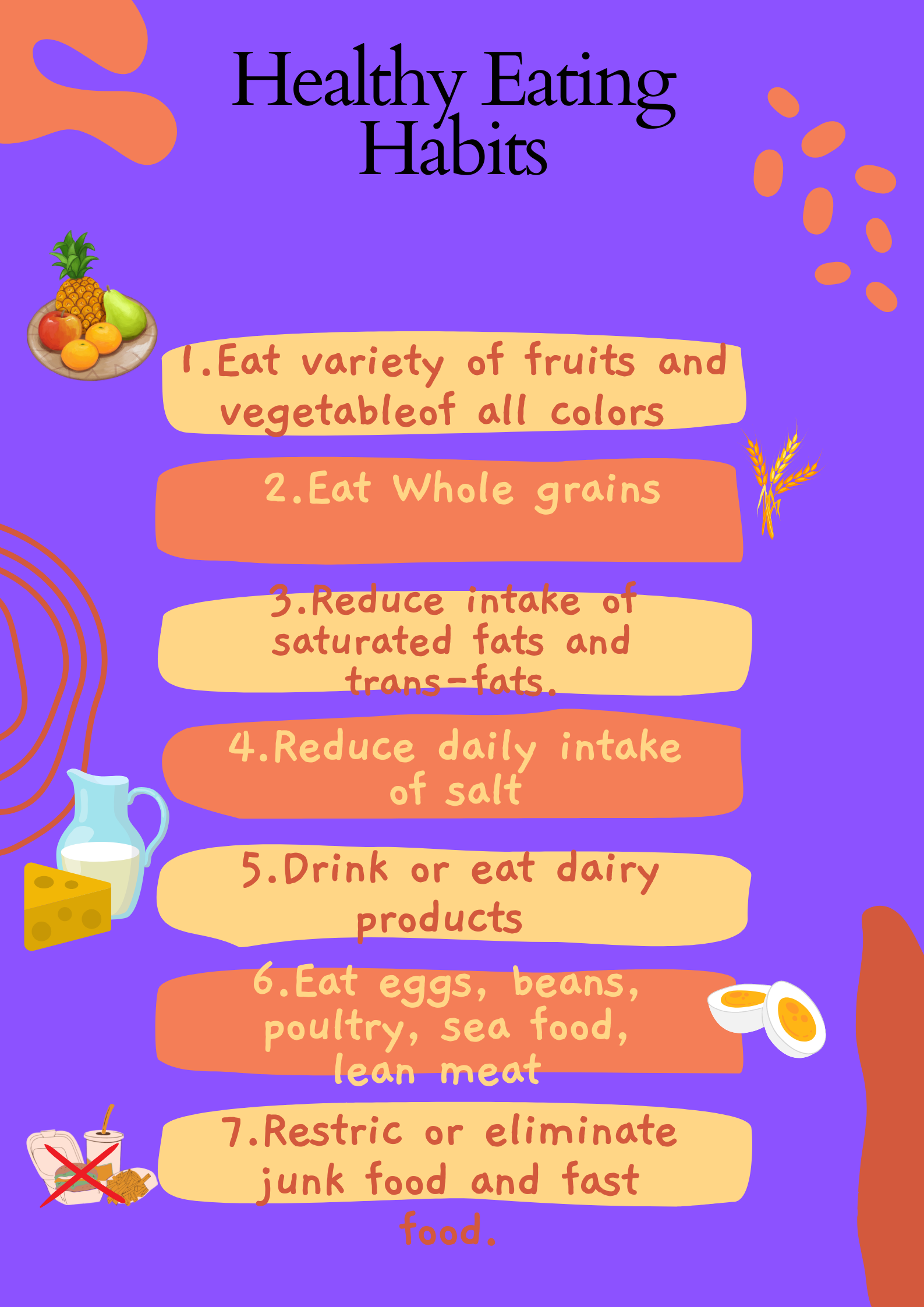
**

**
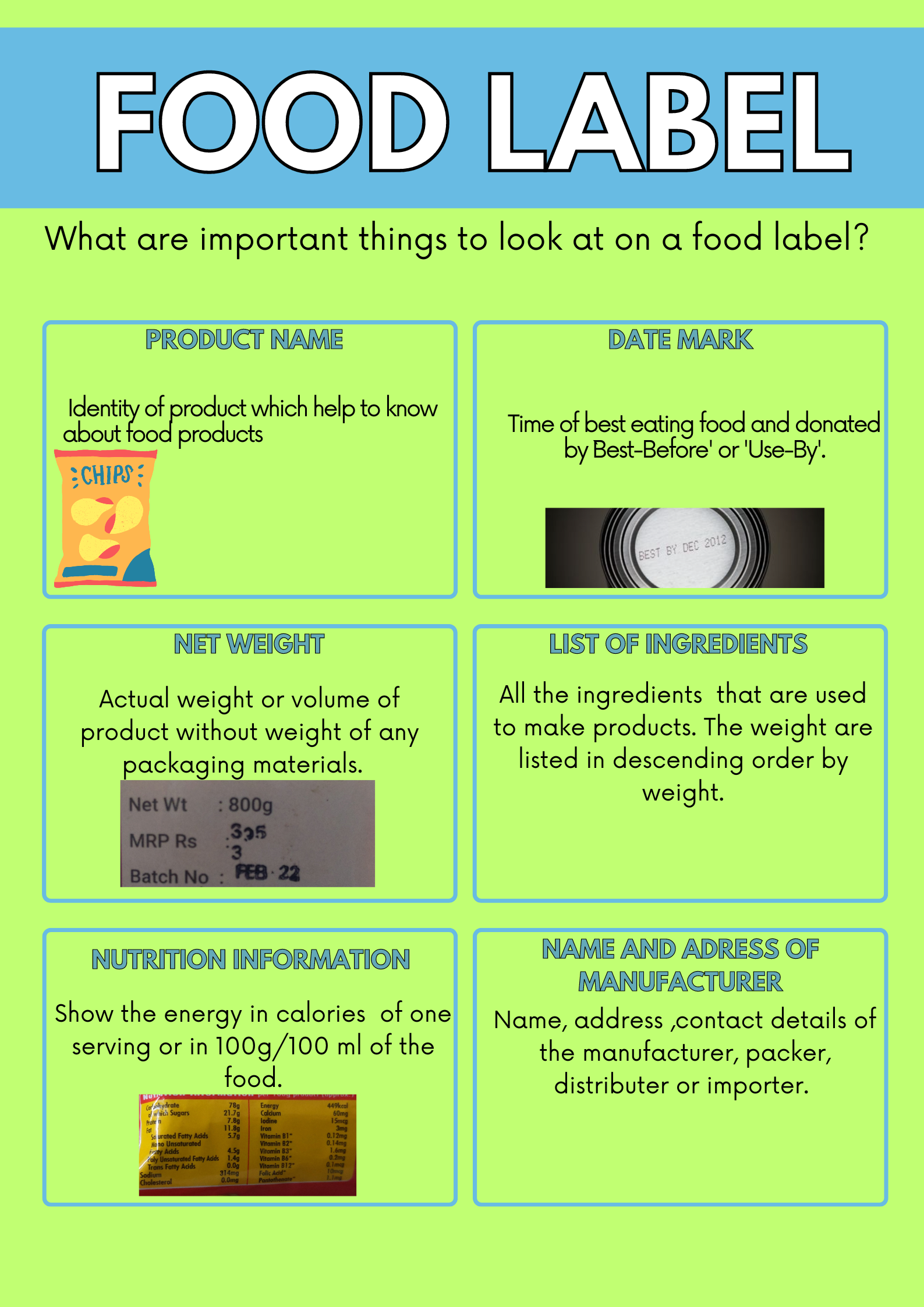
**

**
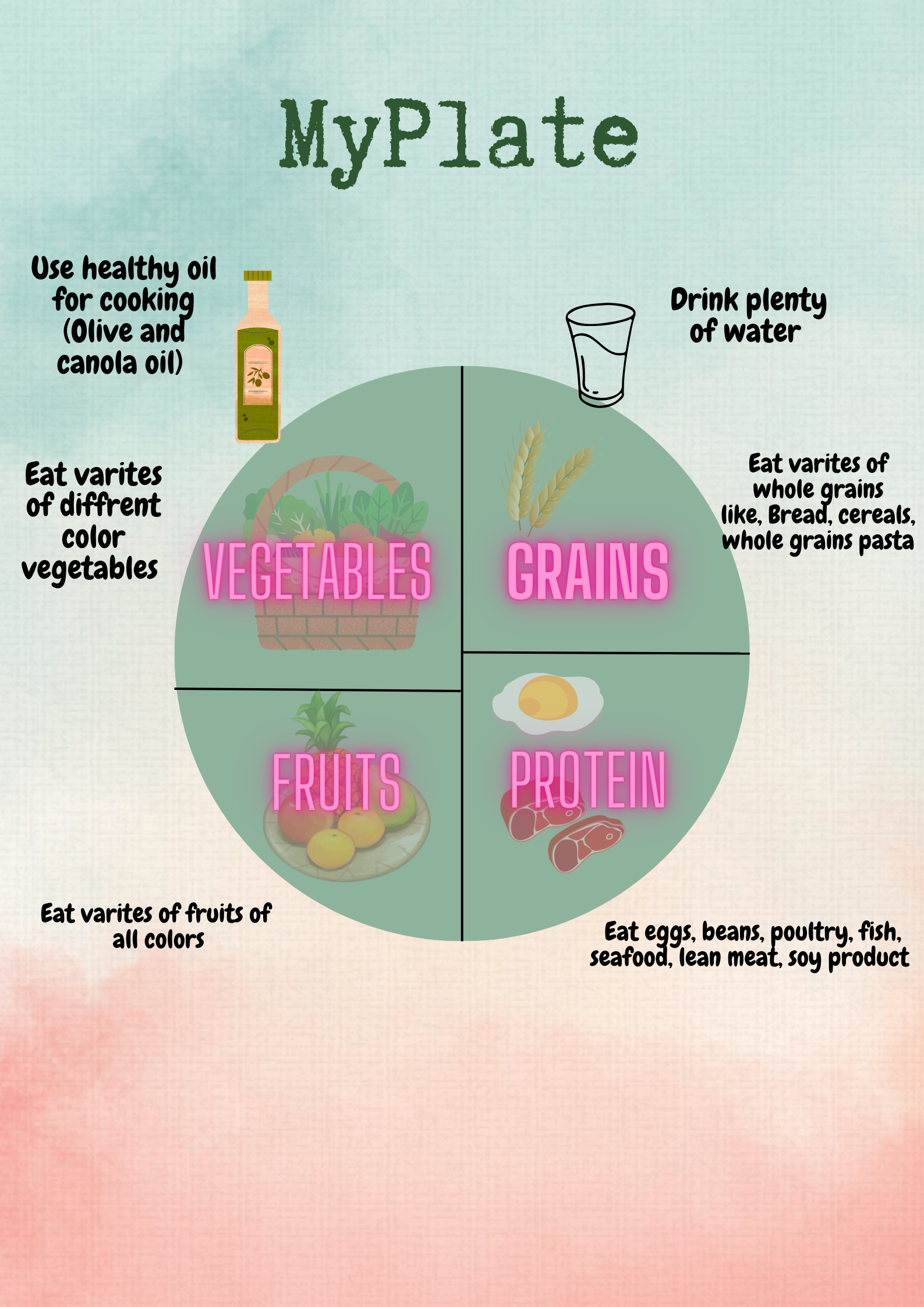
**

**
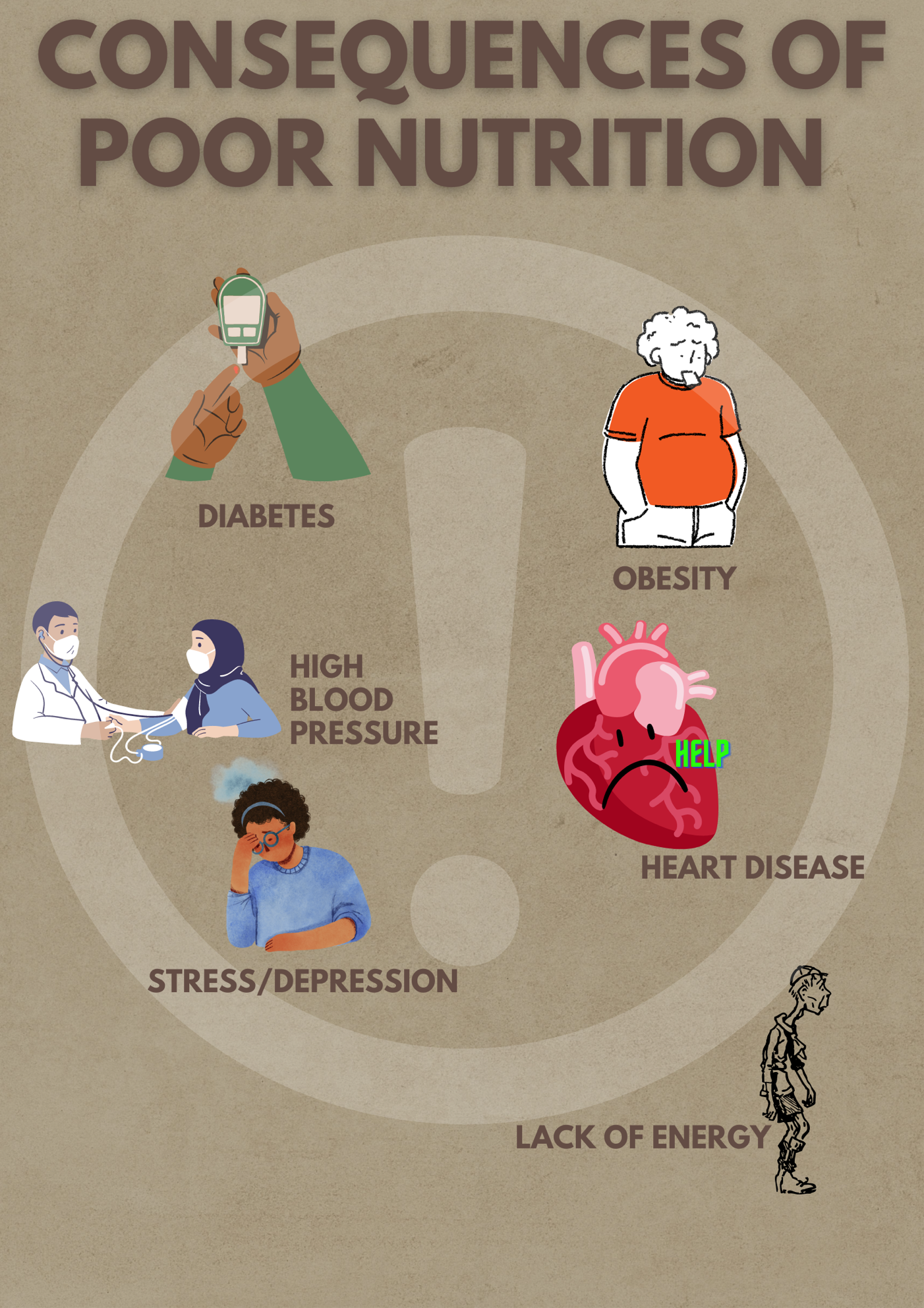
**
